# The cerebellum influences vocal timing

**DOI:** 10.7554/eLife.40447

**Published:** 2018-08-28

**Authors:** Court Hull

**Affiliations:** Department of NeurobiologyDuke UniversityDurhamUnited States

**Keywords:** songbirds, cerebellum, basal ganglia, sensorimotor learning, corticostriatal loops, Area X, Other

## Abstract

A circuit pathway from the cerebellum to the basal ganglia contributes to vocal learning in songbirds.

**Related research article** Pidoux L, Le Blanc P, Levenes C, Leblois A. 2018. A subcortical circuit linking the cerebellum to the basal ganglia engaged in vocal learning. *eLife* **7**:e32167. doi: 10.7554/eLife.32167

Spoken language is a fundamental human skill that relies on vocal learning. Many species are able to produce vocalizations, but only a small number are considered vocal learners. For example, humans learn to speak by imitating the speech of others, and juvenile passerine songbirds learn to sing by mimicking adult birds (Bolhuis et al., 2010).

In both humans and songbirds, the brain circuits essential for vocal learning connect the cortex (a highly evolved brain structure involved in associative learning) and the basal ganglia (a more evolutionarily ancient brain region involved in reinforcement learning; Brainard and Doupe, 2013; Mooney, 2009). In humans, another part of the brain, called the cerebellum or ‘little brain’, may also have an important role in vocal learning. This region is highly active during speech, and children with cerebellar dysfunction often take much longer to learn how to speak (Ziegler and Ackermann, 2017). Moreover, patients with cerebellar disease or damage often suffer from ‘ataxic dysarthria’, a motor speech disorder that affects the timing and clarity of speech (Ackermann, 2008). A better knowledge of how cerebellar circuits interact with the basal ganglia and the cortex is thus critical for understanding how vocal learning is established, and how it is disrupted by injury or disease.

Birds are commonly used to study vocal learning, but the role of the cerebellum in birdsong has so far been unclear. Now, in eLife, Ludivine Pidoux and colleagues at Paris Descartes University report that this structure is also essential for the timing aspects of vocal learning in zebra finches (Pidoux et al., 2018).

Studies in humans and non-human primates have shown that the cerebellum contributes to motor control and motor learning through several pathways. In addition to descending pathways to the spinal cord, the cerebellum connects to the cortex and the basal ganglia via the thalamus, a central structure that relays motor and sensory signals to and from the cortex (Strick et al., 2009; Bostan and Strick, 2018). Since an anatomical connection between the cerebellum and the basal ganglia is also present in songbirds, it was important to test whether the cerebellum could influence the activity of basal ganglia and participate in song learning (Person et al., 2008).

Using a series of elegant anatomical, electrophysiological and pharmacological approaches, Pidoux et al. demonstrate for the first time that this cerebellar pathway has an important role in vocal learning in birds. Stimulating a cluster of neurons in the cerebellum known as the dentate nucleus, activated neurons that participate in song learning within the basal ganglia (Area X) via the thalamus (Figure 1). This activity also propagated via the thalamus to various areas in the cortex, including certain motor areas controlling the vocal chords.

**Figure 1.**
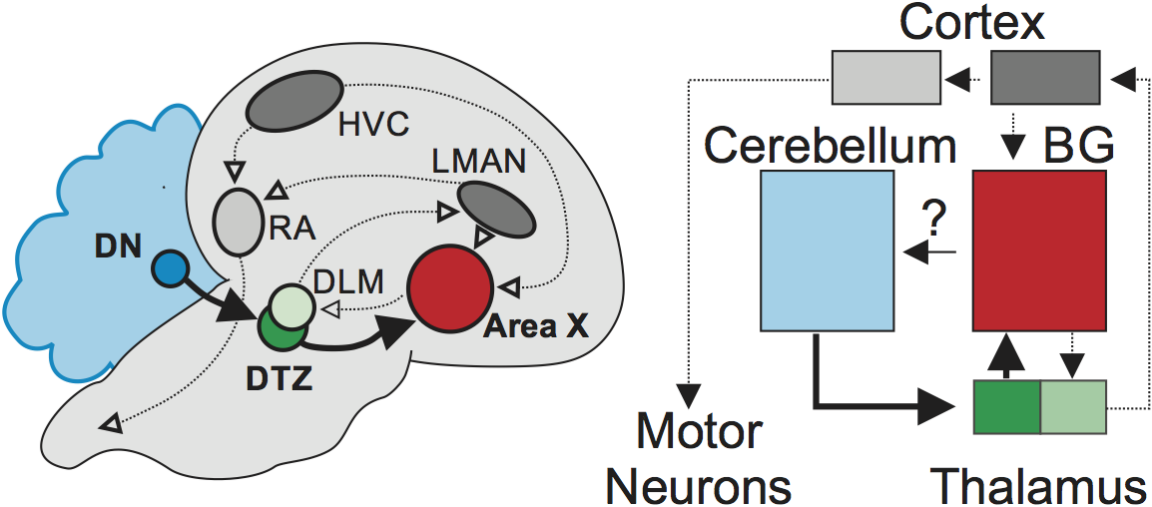
Brain circuits for vocal learning in songbirds and humans. Songbird circuits that support vocal learning (left), also labeled according to their homologous structures in humans (right). These include the cortex (gray and dark gray), the basal ganglia (Area X/BG; red) and the thalamus (green and light green). Pidoux et al. have revealed a functional connection (bold arrows) from an area in the cerebellum, the dentate nucleus (DN; blue), through the dorsal thalamic zone in the thalamus (DTZ; green) to Area X (red) in the basal ganglia. Abbreviations: **DLM** medial portion of the dorsolateral nucleus of the anterior thalamus; **HVC** song-related motor nuclei, used as proper name; **LMAN** lateral magnocellular nucleus of the anterior nidopallium; **RA** robust nucleus of the arcopallium.

This pathway appears to be the only route for the dentate nucleus to modulate the basal ganglia. When the activity was blocked in thedorsal thalamic zone connecting the cerebellum and Area X, neurons in the basal ganglia were prevented from responding to stimulation in the cerebellum. In contrast, blocking the pathways connecting the cortex with Area X did not affect the activity of this region. These results suggest that the cerebellum can influence the circuits in the basal ganglia required for vocal learning, implying that it could play a key role in this process.

Indeed, when this cerebellar pathway was disrupted, juvenile birds were less able to copy the songs of adults. This manipulation particularly affected aspects of song timing. However, this was not the case when the same pathway was disrupted in adult birds, suggesting that the cerebellum is specifically relevant for learning key aspects of song timing. This is consistent with the well-known role of the cerebellum in learned motor timing (Ivry and Spencer, 2004; Mauk et al., 2000). Together, these results provide a complete functional circuit pathway from the cerebellum to the basal ganglia to the premotor neurons involved in song production.

By identifying the specific role of the cerebellum and its circuits in regulating how the timing of a song is learned, Pidoux et al. have shed new light on the neural basis of vocal learning. However, we are only starting to understand how the cerebellum contributes to vocal learning. Next, we need to discover exactly how this brain region shapes the timing of the learned songs. Meanwhile, the ‘little brain’ must be recognized as a key player in the network of circuits that enable vocal learning.
